# Selection of *Bactrocera tau* (Walker) Reference Genes for Quantitative Real-Time PCR

**DOI:** 10.3390/insects16050445

**Published:** 2025-04-24

**Authors:** Yutong Zhai, Yonghao Yu, Pengfei Xu, Xianru Zeng, Xiuzhen Long, Dewei Wei, Zhan He, Xuyuan Gao

**Affiliations:** 1 Plant Protection Research Institute, Guangxi Academy of Agricultural Sciences, Nanning 530007, China; zaak654@163.com (Y.Z.); yxp1127@163.com (Y.Y.); xpfei0306@163.com (P.X.); zxr@gxaas.net (X.Z.); longxiuzhen2006@163.com (X.L.); wdw@gxaas.net (D.W.); happy-hezhan@163.com (Z.H.); 2 Key Laboratory of Green Prevention and Control on Fruits and Vegetables in South China Ministry of Agriculture and Rural Affairs, Nanning 530007, China; 3 Guangxi Key Laboratory of Biology for Crop Diseases and Insect Pests, Nanning 530007, China

**Keywords:** reference genes, RT-qPCR, *Bactrocera tau*, different stages, different body parts

## Abstract

The selection of appropriate reference genes is critical for standardizing quantitative real-time reverse transcription polymerase chain reaction (RT-qPCR) data, thereby ensuring accurate and reliable results of gene expression analysis. In this study, we identified 10 candidate reference genes (encoding *α-tubulin*, *G6PDH*, *Rab1*, *RT*, *RPS13*, *β-tubulin*, *DPH1*, *HSP90*, *GAPDH*, and *CP*) and evaluated their suitability for use as reference genes in the pest insect, *Bactrocera tau*. Analysis was conducted using three software-based methods—Delta CT, NormFinder, and BestKeeper—alongside the online tool RefFinder. Expression levels of these genes were analyzed across various *B. tau* developmental stages and body parts. The overall ranking of reference gene stability scores was as follows: *α-tubulin* > *G6PDH* > *CP* > *β-tubulin* > *RT* > *HSP90* > *GAPDH* > *DPH1* > *RPS13* > *Rab1*. Ultimately, *α-tubulin* and *G6PDH* were identified as the most stable reference genes for *B. tau*.

## Simple Summary

Scientists often need to make gene expression analysis results accurate, and a key part of this is choosing the right reference genes. In this research, the focus was on the pest insect *Bactrocera tau*. Ten candidate reference genes were selected and analyzed based on the transcriptome sequencing data of *B. tau*. To figure out which ones were the best, three software methods, called Delta CT, NormFinder, and BestKeeper, were used along with an online tool named RefFinder. We studied how these genes were expressed at different life stages of *B. tau* and in various body parts. After careful analysis, the genes were ranked according to their stability. The top two most stable genes were the ones that code for *α-tubulin* and *G6PDH*. This discovery is important because it will help other scientists conduct more accurate gene expression studies on *B. tau*. This, in turn, can aid in better understanding and controlling this pest, which benefits society by potentially reducing crop damage and economic losses related to the pest.

## 1. Introduction

*Bactrocera tau* (Walker) is a globally significant agricultural pest insect, prevalent in South and Southeast Asia, and particularly in southern regions of China [1,2]. *B. tau* is characterized by a prolonged life cycle and has a capacity for multiple ovipositions. It exhibits a wide range of feeding habits that contribute to the decay of fruits and vegetables, resulting in substantial economic losses [3]. Furthermore, *B. tau* is an invasive species that poses a considerable threat to crops in numerous countries, including Pakistan, Japan, Indonesia, and the United States [4]. Currently, chemical methods predominate among methods used for population management of *B. tau*, and involve strategies including male attraction via phenylbutanoids and Cue-lure (4-(4-acetoxyphenyl)-2-butanone) [5,6]; however, control of *B. tau* remains challenging because of the ability of females to lay eggs multiple times after a single mating, coupled with their high population densities.

With the completion of chromosome assembly, Iso-Seq, and RNA-seq analyses of *B. tau* [2,7], there has been a growing interest in elucidating the functions of genes in this species. For example, Zhang et al. employed quantitative real-time reverse transcription polymerase chain reaction (RT-qPCR) and RNA interference technologies to investigate the role of the phenoloxidase (PO) gene in melanin biosynthesis during insect development post-sequencing [8]. RT-qPCR is an indispensable tool in gene research, widely used to characterize gene expression across different tissue and cell types, and during various developmental processes [9,10]. Two primary quantification methods, absolute and relative, are used to measure gene expression by RT-qPCR. Among them, the latter approach is predominant [11]. Use of suitable reference genes in RT-qPCR data analysis is essential for minimizing the impact of errors and enhancing the accuracy and reliability of experimental results [12,13]. Moreover, in scenarios involving numerous samples or significant biological variation, comprehensive assessment, using the geometric mean of multiple reference genes, is necessary to ensure analytical precision [14,15].

Reference genes are defined as genes that demonstrate stable transcription across various cells or physiological states [16]. During selection of reference genes, it is crucial to ensure their expression remains stable under diverse conditions; however, empirical analyses have revealed a general dearth of universally stable reference genes. Expression levels can fluctuate due to variations in species, developmental stage, and cell type, among other factors [4,17,18,19,20,21]. Furthermore, even genes frequently regarded as reference genes may yield inconsistent results under varying experimental conditions [22].

Consequently, to guarantee the accuracy of experimental outcomes, it is imperative to assess the expression stability of each candidate reference gene under defined experimental conditions. Common reference genes, such as *β-Actin* and *EF-α*, have been selected to evaluate the expression levels of prophenoloxidase-1 and PO throughout the *B. tau* life cycle, as reported by Liu et al. [7]; however, no prior studies have documented the stability of *β-Actin* and *EF-α* expression from larvae to pupae in *B. tau*, nor have there been investigations using high-throughput sequencing methods to screen reference genes across different *B. tau* developmental stages and body parts.

In this study, based on indicators such as the coefficient of variation in genes and the Fragments Per Kilobase of exon per Million reads mapped (FPKM) values, and referring to the commonly used and widely recognized types of reference genes in relevant research, we screened a total of 10 candidate reference genes from the transcriptome sequencing data of the testes and accessory glands of male *B. tau* (Zhai et al., unpublished); specifically, genes encoding *α-tubulin*, *G6PDH*, *Rab1*, *RT*, *RPS13*, *β-tubulin*, *DPH1*, *HSP90*, *GAPDH* and *CP* were analyzed. A series of RT-qPCR experiments were conducted to evaluate the stability of these genes across various developmental stages and body parts. Candidate reference gene stability was assessed using three computational methods—Delta (Δ) Ct, BestKeeper, and NormFinder—in conjunction with the online tool, RefFinder. The two most stable genes are proposed as reliable reference genes for investigating gene expression across different developmental stages and body parts of *B. tau*. The findings of this study provide valuable data regarding reference genes for use in *B. tau* gene expression analysis and establish a theoretical foundation from which to explore gene functions. By employing validated reference genes, researchers can enhance their understanding of differential gene expression throughout various life cycle stages and body parts, facilitating improved insights into the biological characteristics of *B. tau* and enabling more effective control measures.

## 2. Materials and Method

### 2.1. Biological Samples

*B. tau* larvae were collected from the Luohan orchard in Xinglong Village, Longjiang Township, Yongfu County, Guilin City, Guangxi, and subsequently housed in the insect rearing facility of the Plant Protection Institute, Guangxi Academy of Agricultural Sciences, where they were raised on *Siraitia grosvenorii*. On maturation, larvae were individually transferred into glass bottles containing sand for pupation. Following eclosion, adults were relocated to rearing cages, measuring 35 cm in length, width, and height, and fed a diet consisting of equal parts yeast powder and sucrose.

A total of 14 experimental samples were collected, including eggs, first to third instar larvae, 1-day-old pupae, 7-day-old pupae, newly emerged males and females, and fully mature (13-day-old) males and females, as well as adult head, thorax, male abdomen, and female abdomen specimens, with 3 biological replicates for each sample. After collection, samples were rinsed with pre-cooled 0.1% PBS, and body parts were dissected in 0.1% PBS solution, followed by extensive washing in the same solution, before transfer to 1.5 mL sterile centrifuge tubes. Samples were then rapidly frozen in liquid nitrogen and stored at −80 °C. Experiments comprised three biological replicates, and sampling data for each replicate are presented in Table 1.

### 2.2. Total RNA Extraction and cDNA Synthesis

Total RNA samples were extracted using Shanghai Bioengineering RNA Simple Isolation Extraction Reagent. Extracted total RNA quality and concentration were assessed using a micro-volume ultraviolet spectrophotometer (NanoDrop-2000c, Thermo Fisher Scientific (Waltham, MA, USA)), with RNA purity confirmed by calculating the 260/280 nm absorbance ratio, which should ideally be approximately 2.0. RNA integrity was evaluated by denaturing agarose gel (1%) electrophoresis. Aliquots of qualified RNA samples (1 μg) were used as template for first-strand cDNA synthesis with a TransScript^®^ All-in-One First-Strand cDNA Synthesis SuperMix for qPCR (One-Step gDNA Removal) reverse transcription kit. All procedures were conducted in accordance with the manufacturer’s instructions.

### 2.3. Primer Design and Sequence Accuracy Validation

Reference gene sequences corresponding to various biological processes were selected from the *B. tau* transcriptome database, including genes encoding *Rab1*, *RT*, *RPS13*, *β-tubulin*, *DPH1*, *CP*, *HSP90*, *GAPDH*, *α-tubulin*, and *G6PDH*. The sequences were amplified by RT-PCR. The PCR products were sent to Shanghai Sangon Biotech Co., Ltd (Shanghai, China). for sequencing to verify the accuracy of each candidate reference gene sequence. The RT-PCR reaction conditions were as follows: an initial denaturation at 95 °C for 3 min, followed by 35 cycles of denaturation at 95 °C for 30 s, annealing at 58 °C for 30 s, and extension at 72 °C for 1 min, with a final extension at 72 °C for 10 min. Primers used for RT-PCR and RT-qPCR during the study were designed using Primer 5.0 software.

### 2.4. RT-qPCR and Amplification Efficiency Testing

RT-qPCR assays were conducted in a total volume of 20 μL, comprising 10 μL 2× ChamQ Universal SYBR qPCR Master Mix, 0.4 μL each of forward (F) and reverse (R) primers (10 μM), 2 μL cDNA template, and 7.2 μL sterile double-distilled water. Reaction conditions were implemented according to the instructions provided with the fluorescence quantitative PCR reagent. Following reaction completion, melting curve analysis was performed to assess primer specificity based on melting curve peak profiles. cDNA template samples were serially diluted by a factor of 2, yielding concentrations of 2^0^, 2^−^^1^, 2^−^^2^, 2^−^^3^, 2^−^^4^, and 2^−^^5^ ng/μL, followed by qPCR. The resulting data were processed, and logarithmic values obtained by setting the logarithm of the cDNA template concentration (log) as the horizontal coordinate and cycle threshold (Ct) values as the vertical coordinate. Data were subjected to regression analysis using SPSS 24 software, to determine the linear equation, along with the correlation coefficient (R^2^) and slope variables. Amplification efficiency (E) was subsequently calculated using the specific expression formula: E = (10^(−1/slope)^ − 1) × 100. Each reaction was performed in triplicate.

### 2.5. Verification of the Stability of Reference Genes

To verify the stability of reference genes, second-instar larvae of *B. tau* with uniform growth status and size were selected. Larvae were placed in sterilized Petri dishes containing *Siraitia grosvenorii* and incubated at constant temperatures (0, 10, 20, 30, 40 °C) for 1 h in a temperature-controlled incubator, with seven larvae per sample and three biological replicates. Samples were immediately frozen in liquid nitrogen for subsequent analysis. The most stable genes, which were screened through comprehensive software-based analysis, were utilized as reference genes. The RT-qPCR method was consistent with that described in Section 2.4.

### 2.6. Statistical Analysis

Candidate genes were amplified under optimal conditions using an RT-qPCR instrument and average Ct values were calculated from three biological replicates. Ct values obtained by RT-qPCR were subjected to variance analysis using SPSS 24 software. Data were then analyzed using Delta(Δ) Ct, NormFinder [23], and BestKeeper [14] methods to evaluate the stability of the ten candidate reference genes under varying conditions. Ct values were converted to relative expression levels using the 2^−ΔCt^ method, where ΔCt represents the difference between the Ct values of all samples under identical conditions and the smallest Ct value. The ΔCt method is predicated on the principle that gene stability is inversely proportional to the standard deviation (SD) of ΔCt values, with gene expression stability ranked by comparison of the SD values of candidate gene ΔCt values. Subsequently, 2^−ΔCt^ values were calculated for analysis using NormFinder, which assesses the stability value (SV) of genes by comparing within- and between-sample variation, and ranking genes based on SV values, to evaluate gene stability; this software is distinguished by its capacity to compute variations between samples and select the most suitable reference gene. BestKeeper facilitates the comparison of expression levels across up to ten reference genes and ten target genes, automatically calculating SD and *p* values from Ct value inputs, to ascertain reference gene stability; a smaller SD indicated greater stability and if *p* > 0.05, the result was deemed statistically insignificant. This software was used to analyze the expression levels of target genes. The RefFinder online tool was applied for the synthesis and comparison of results and the ultimate selection of the most stably expressed genes under each condition as final reference genes. Charts were generated using OriginPro 2021b SR1 v9.8.5.204 x64.

## 3. Results

### 3.1. Selection of Candidate Reference Genes

**Figure 1:**
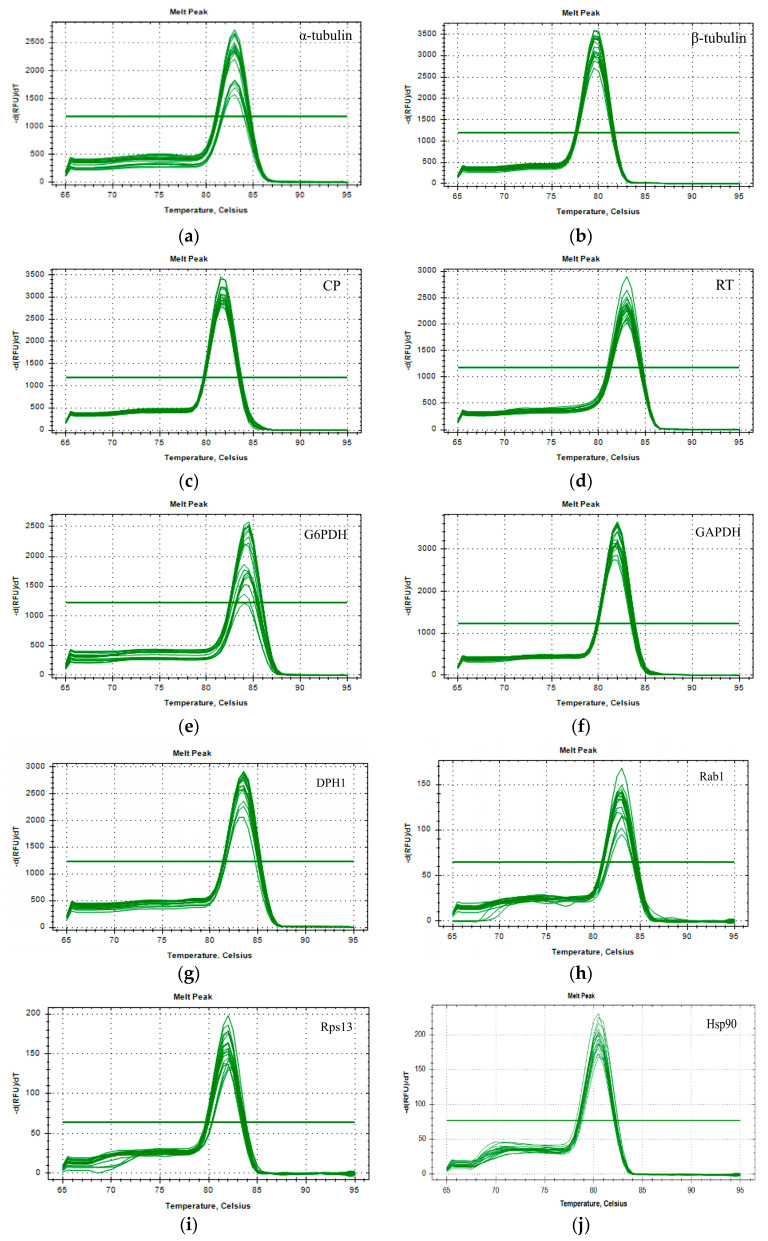
Melting curve analyses of ten candidate *Bactrocera tau* reference genes of for use in RT-qPCR: (**a**) *α-tubulin*; (**b**) *β-tubulin*; (**c**) *CP*; (**d**) *RT*; (**e**) *G6PDH*; (**f**) *GAPDH*; (**g**) *DPH1*; (**h**) *Rab1*; (**i**) *RPS13*; (**j**) *HSP90*.

The ten candidate reference genes were amplified by RT-PCR using the primers detailed in Table 2. Sequencing results of the PCR products showed 100% consistency with transcriptome sequencing data, confirming the accuracy of the selected gene sequences and ensuring reliability of subsequent experimental results. The usability of designed qPCR primers was validated through RT-qPCR. As shown in Figure 1, each candidate reference gene produced a single peak in the melting curve analysis, demonstrating excellent specificity of the selected primers. Amplification efficiency (E) and regression coefficient (R^2^) values were calculated based on the slopes of standard curves generated for each primer pair. E-values ranged from 93.286% (*DPH1*) to 108.880% (*α-tubulin*), all falling within the acceptable range of 80.0–120.0% (Table 3).

### 3.2. Variation in Expression of the Ten Reference Genes

**Figure 2:**
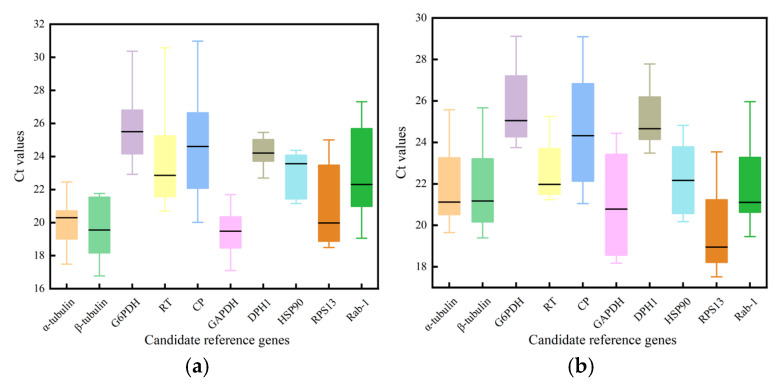

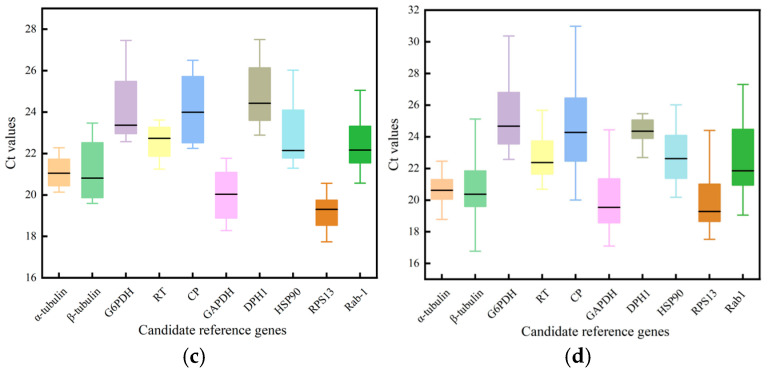
Range of all Ct values for ten candidate *Bactrocera tau* reference genes in different samples: (**a**) development stages before eclosion; (**b**) adults; (**c**) body parts; (**d**) all samples.

The expression levels of the ten candidate reference genes were also analyzed by RT-qPCR, with the resulting Ct values reflecting gene expression levels under various conditions. Results showed that during the pre-eclosion developmental stages, *α-tubulin* and *β-tubulin* exhibited smaller Ct-value variations and higher expression stability (Figure 2a). In the adult stage, *RT* and *DPH1* exhibited smaller Ct-value variations and higher expression stability, but *DPH1* had larger Ct values, indicating lower gene-expression levels (Figure 2b). In different body parts, *α-tubulin*, *RT*, and *RPS13* exhibited smaller Ct-value variations and higher expression stability (Figure 2c). The range of mean Ct values was 20.01 (*GAPDH*) to 25.02 (*DPH1*) across all samples, with *α-tubulin* and *DPH1* exhibiting the least variation in expression, while greater variability was observed in the levels of *CP* and *Rab1* (Figure 2d).

### 3.3. Stability of Expression of the Ten Reference Genes in Various Samples

**Figure 3:**
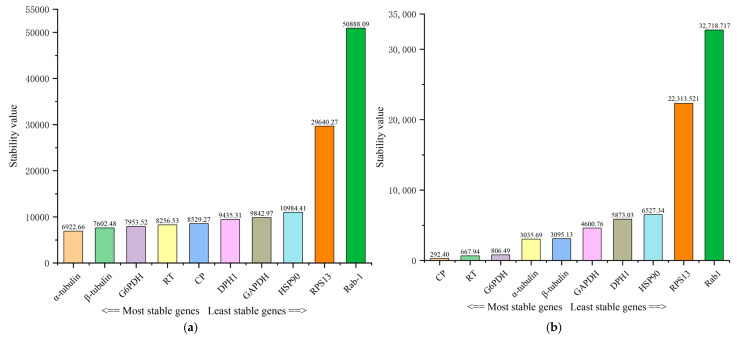

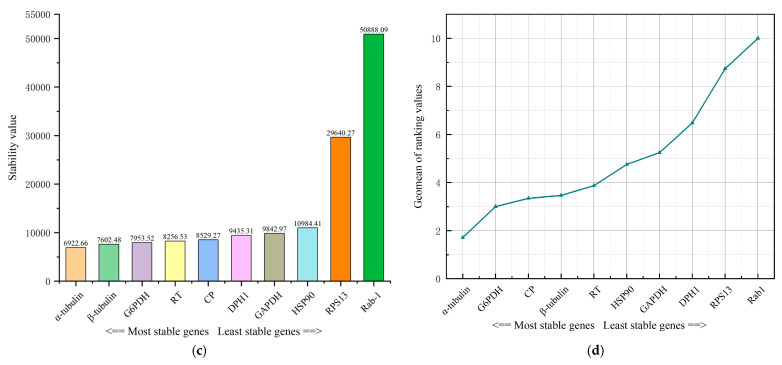
The stability of 10 candidate reference genes was evaluated using RefFinder, an integrated tool combining Delta CT, BestKeeper, and NormFinder algorithms: (**a**) gene stability assessment using the Delta CT method; (**b**) gene stability assessment using the BestKeeper method; (**c**) gene stability assessment using the NormFinder method; (**d**) comprehensive gene stability assessment using RefFinder.

Candidate reference gene stability was assessed using the ΔCt, BestKeeper, and NormFinder methods, as well as the RefFinder online tool. Based on ΔCt analysis, *α-tubulin*, *β-tubulin*, and *G6PDH* were the most stable reference genes across various *B. tau* developmental stages and body parts, with gene SD values all <15,000. In contrast, *Rab1* emerged as the most unstable reference gene, with SD values as high as 51,769.88 (Figure 3a). According to BestKeeper analysis, *CP* was the most stable reference gene (SV < 300), while *Rab1* remained the most unstable reference gene (Figure 3b). NormFinder analysis further corroborated these findings, revealing that *α-tubulin*, *β-tubulin*, and *G6PDH* demonstrated robust stability, with SV all <8000, while *Rab1* continued to rank as the least stable reference gene (Figure 3c). Comprehensive scoring of the stability of the ten candidate reference genes across different *B. tau* developmental stages and body parts, using the RefFinder online tool, yielded the following ranking from highest to lowest stability: *α-tubulin* > *G6PDH* > *CP* > *β-tubulin* > *RT* > *HSP90* > *GAPDH* > *DPH1* > *RPS13* > *Rab1* (Figure 3d). Consequently, we recommend use of *α-tubulin* and *G6PDH* as the optimal combination of reference genes for quantifying target gene expression across various developmental stages and body parts in *B. tau*.

### 3.4. Empirical Verification of Reference Gene Stability

**Figure 4:**
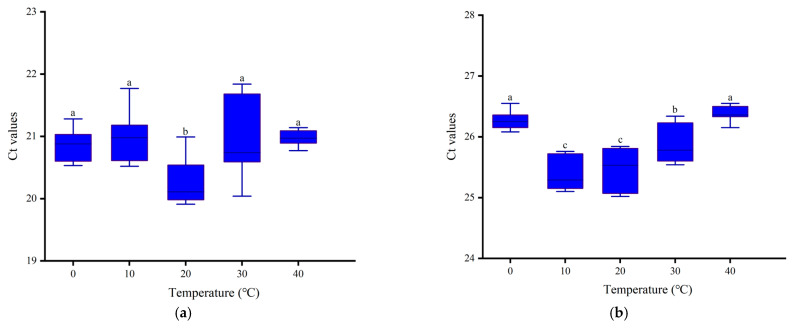
Verification of the stability of reference genes: (**a**) expressions of *α-tubulin* in 2nd instar larvae treated at different temperatures; (**b**) expressions of *G6PDH* in 2nd instar larvae treated at different temperatures. Note: Different lowercase letters indicate significant differences (*p* < 0.05).

We selected *α-tubulin* and *G6PDH*, which exhibit high expression stability, and conducted verification using second-instar larvae of *B. tau* treated at different temperatures as samples. Under the treatment at 0–40 °C, the Ct values of the α-tubulin gene ranged from 19.91 to 21.84 (Figure 4a), while those of the G6PDH gene ranged from 25.02 to 26.55 (Figure 4b). In different temperature treatment groups, the differences in the transcriptional level expressions of *α-tubulin* and *G6PDH* were generally small, and all the difference values were within 2. These results demonstrate that the gene expressions of *α*-*tubulin* and *G6PDH* remain stable under environmental stress, qualifying them as suitable reference genes for gene expression studies in *B. tau*.

## 4. Discussion

Selection of appropriate reference genes is essential for the analysis of target gene expression levels; however, reference genes may vary within the same species based on environmental factors, developmental stage, and tissue type, and identifying universally applicable reference genes remains a significant challenge [20,24,25,26]. In this study, we employed the ΔCt, BestKeeper, and NormFinder methods, in conjunction with the RefFinder online tool, to conduct a comprehensive analysis of the stability of ten candidate reference genes across distinct developmental stages and various male and female adult body parts of *B. tau*.

We first carried out a group-based study on the stability of reference genes at different stages. The candidate reference genes with stable expression varied among different groups, and it is speculated that this is related to the different life activities of *B. tau* at various growth stages. Since the development stages before eclosion are periods when the insect experiences drastic changes in morphology and physiological activities, accompanied by frequent cellular activities, in order to ensure the smooth progress of the development process, genes involved in the construction of the cytoskeleton, such as *α*-*tubulin* and *β*-*tubulin*, are stably expressed. In the adult stage, *RT* and *DPH1* exhibited high expression stability. The physiological activities and functions of insects in the adult stage differ significantly from those in the larval stage, such as reproduction, feeding, and flying. *RT* and *DPH1* may be involved in the physiological processes specific to adults, and their stable expression is crucial for maintaining the normal operation of these physiological functions. Among different body parts, *α*-*tubulin*, *RT*, and *RPS13* showed high expression stability. Different body parts have distinct tissue compositions and physiological functions. It is likely that these three genes are involved in some basic cellular processes that are widely present in various tissues, such as protein synthesis (*RT*, *RPS13*) and cytoskeleton maintenance (*α*-*tubulin*). In the future, more detailed and in-depth research on the selection of reference genes at different stages can be carried out according to specific needs. In the subsequent part of this study, the reference genes throughout the entire life cycle and in different tissues of *B. tau* will be mainly analyzed using the Delta CT, NormFinder, BestKeeper, and RefFinder methods.

The results from ΔCt, BestKeeper, and NormFinder were not entirely consistent. The findings generated using the ΔCt and NormFinder methods were very similar, with both identifying *α-tubulin*, *β-tubulin*, and *G6PDH* as the top three most stable reference genes, whereas BestKeeper ranked *CP*, *RT*, and *G6PDH* as the top three. These discrepancies arise from the unique algorithms employed by each method. To address the limitations inherent to any single method, the RefFinder online tool was applied to calculate the geometric mean of the stability rankings obtained from the three analytical approaches, leading to a comprehensive ranking. The top two genes identified were *α-tubulin* and *G6PDH*.

Both α-and *β-tubulin* exist in cells as dimers and serve as fundamental components of microtubules [27], which are integral to intracellular transport, cell division, and cell motility, among other essential functions [28]. *G6PDH* is a highly conserved enzyme with a pivotal role in glucose homeostasis and the pentose phosphate pathway, which also influences redox metabolism and significantly affects cell growth [29]. Given their critical roles in cell activities, these genes are frequently used as reference genes in various species. For example, T. Liu et al. (2021) identified *α-tubulin* as the most stable gene under cold treatment and *G6PDH* as the most stable gene under heat treatment in *Bactrocera dorsalis* [30]; however, these genes are not universally suitable as reference genes across all species. Shen et al. (2022) demonstrated that *α-tubulin* and *G6PDH* were not optimal reference genes under three different experimental conditions (developmental stages, tissues/organs and temperatures) in *Phthorimaea operculella* [22]. Similarly, Silveira et al. (2021) found that, among 25 candidate reference genes in *Schistosoma mansoni*, *α-tubulin* and *G6PDH* ranked as the least stable [11]. Hence, the expression stability of the same genes can fluctuate significantly according to species, tissue, and treatment, underscoring the importance of exploring *B. tau* reference genes for use in research studies in various developmental stages and body parts.

Ribosomal proteins are essential for ribosome assembly and protein translation and participate in diverse physiological and pathological processes [31]. Lü et al. summarized trends in reference gene selection for insect gene expression studies from 2008 to 2017 and noted that ribosomal protein genes were the most commonly chosen reference genes in insect gene expression research [32]. In this study, we found that *RPS13* was unsuitable as a reference gene in *B. tau*, due to its inadequate stability across the three analytical methods used and low ranking on assessment using RefFinder, indicating that it is among the most unstable genes in various *B. tau* developmental stages and body parts. Consistent with our findings, Shen et al. (2023) found that *RPS13* was not a suitable reference gene in *Aphidoletes aphidimyza* [33].

*Rab1* proteins are crucial regulators of intracellular membrane transport, involved in the formation and transport of secretory proteins [34,35], which also has a role in mitosis and meiosis in *Drosophila melanogaster* [36]. In this study, *Rab1* consistently ranked lowest across all three analytical methods and received the lowest comprehensive score, indicating it is the most unstable gene across different *B. tau* developmental stages and body parts. RT-qPCR data revealed that *Rab1* gene expression was highest during the larval to pupal transition and lowest at the end of pupation, exhibiting significant fluctuations at other stages and in various body parts. We hypothesize that *Rab1* is highly expressed during the larval to pupal stages, when *B. tau* undergoes considerable morphological changes and active cell division occurs. At the conclusion of pupation, when metabolic activity is minimal and extensive cell proliferation is unnecessary, *Rab1* expression diminishes. Consequently, this gene is unsuitable as a reference for use in *B. tau* gene expression studies. Similarly, during the process of screening reference genes for *Varroa mites*, Lee J also found that *Rab1* showed the least stable expression patterns both after acaricide treatments and across different tissues [37].

This study shows that *α-tubulin* and *G6PDH* are the most stable reference genes for *B. tau* under non-environmental stress conditions. However, there are certain limitations in their application. In order to verify the stability of *α-tubulin* and *G6PDH* under environmental stress conditions, we conducted qRT-PCR using the second-instar larvae of *B. tau* treated with different temperatures. All the difference values were within 2, which indicates that *α-tubulin* and *G6PDH* can also serve as reliable reference genes for *B. tau* under stress induction [38]. Ponton F et al. found in their study of reference genes in *Drosophila melanogaster* under conditions such as injury, heat stress, or feeding with different diets that *EF1*, *Actin*, *Rpl32*, and *Tubulin* are the reference genes with the most stable expression in *Drosophila melanogaster* [24]. The study by Lü et al. showed that *G6PDH* and *RpL32* are the reference genes with the most stable expression in *Bactrocera* (Tetradacus) *minax* at different developmental stages and under conditions of temperature stress and γ-ray radiation stress [39]. *Tubulin* and *G6PDH* can be used as reference genes for *Drosophila melanogaster* and *Bactrocera* (Tetradacus) *minax* under different stress inductions, respectively. These results suggest that *α-tubulin* and *G6PDH* may have a certain degree of universality in the screening of reference genes for pests of the Tephritidae family, and can be regarded as one of the reliable reference gene choices for gene expression studies in this group of pests.

Although RefFinder integrates multiple methods and enhances the reliability of the evaluation of the stability of reference genes, this method still has certain limitations. The weight assignment of different algorithms can affect the results. For example, ΔCt is based on relative expression levels, while NormFinder is based on variance, and these differences may lead to deviations in the final screening results. In addition, RefFinder relies on the individual analysis results of each method and cannot completely eliminate the influence of the inherent limitations of each method on the final results. To overcome the above-mentioned issues and improve the accuracy and reliability of the research, future studies may consider combining the absolute quantitative digital PCR technology to validate the screened reference genes. Digital PCR can achieve nucleic acid quantification at the single-molecule level, and compared with traditional PCR technology, it has higher sensitivity and accuracy. By using digital PCR to absolutely quantify the expression levels of reference genes in different samples, their stability can be evaluated more accurately, which can make up for the deficiencies of RefFinder in terms of algorithms and technology, thus providing a more reliable basis for the screening of reference genes in *B. tau*.

In conclusion, our findings indicate that *α-tubulin* and *G6PDH* represent the most stably expressed genes across different *B. tau* developmental stages and body parts, suggesting their suitability as reference genes for quantifying target gene expression. This is the first study to analyze and select candidate reference genes based on exploration of *B. tau* transcriptome sequencing data from various developmental stages and body parts. Our results provide a foundation for molecular research on *B. tau*. By using the two selected internal reference genes, it is possible to explore the expression levels of genes related to male seminal proteins during the mating process of *B. tau*, or genes related to immunity in response to chemical and biological pesticide applications. Additionally, through RNA interference technology, highly expressed genes can be silenced to control the *B. tau* population and increase its mortality rate. However, this study has certain limitations. The applicability of *α-tubulin* and *G6PDH* as reference genes in *B. tau* under different temperature stress conditions, distinct developmental stages, and diverse host plant feeding conditions, as well as other physiological states or experimental conditions, warrants further investigation. Moreover, this study can facilitate the selection of internal reference genes under these conditions. In future research, additional candidate reference genes can be identified based on this study.

## Figures and Tables

**Table 1:** *Bactrocera tau* samples collected.

Samples		Quantity
Development stages before eclosion	Eggs	15
	1st instar larvae	15
	2nd instar larvae	7
	3rd instar larvae	3
	1-day pupae	3
	7-day pupae	3
Adults	Newly eclosed females	2
	Newly eclosed males	2
	13-day females	2
	13-day males	2
Body parts	13-day adult heads	5
	13-day adult thoraxes	3
	13-day female abdomens	3
	13-day male abdomens	3

**Table 2:** Primers to amplify ten candidate reference genes for RT-PCR.

Gene	Primers (5′–3′)	Amplicon Length (bp)
*RPS13*	F: CTGTAAAGATGGGTCG	931
	R: CTGCCACAAATTAACAAC	
*G6PDH*	F: TGCCACTTGTAAGACCC	1458
	R: ACACTTCGATTCCCATG	
*GAPDH*	F: TAAGTAACCGGAGGCT	1636
	R: GAAGGTCGTATCTAATGC	
*α-tubulin*	F: ACGCTGCCAACAACTAT	1074
	R: TTCATTTCGCCGTTTA	
*β-tubulin*	F: CGATTTCGCCGTTAC	1053
	R: GACTCTGTGCTCGATGTT	
*HSP90*	F: TGGTACAAAGGCATTCA	1823
	R: AAGCATCCTCGGTGTC	
*CP*	F: CTTCTATGCCTGGTATGAC	1124
	R: AATCCTCTTCCGTTGG	
*DPH1*	F: TGCCAATGACGATGA	1021
	R: GCCAGTGCCTCCTAT	
*Rab1*	F: TGCGTGGAAGTGAGA	1039
	R: TATTCAGCAGCAACCT	
*RT*	F: GTGCGTAGGGTTTGGG	1428
	R: GCAGGTCGGAGAAGTGTT	

**Table 3:** Primers for ten candidate reference genes used in RT-qPCR.

Gene	Primers (5′–3′)	Amplicon Length (bp)	Slope	Correlation Coefficient (R^2^)	Amplification Efficiency (E) (%)
*α-tubulin*	F: ACGCCTGCTGGGAATTGTACTG	90	−3.126	0.991	108.880
	R: AATCATCACCACCGCCGACAG				
*β-tubulin*	F: GTGGAATATCGCAAACGGCAGTC	120	−3.157	0.997	107.375
	R: GTGGTCGCATGTCAATGAAGGAAG				
*CP*	F: TGAATGGCACCGCTGAGAAT	140	−3.035	0.991	113.545
	R: ACGCTTTTCACCGATCACCT				
*RT*	F: AGGCTGACATCACCGCAATCC	123	−3.042	0.994	113.173
	R: CCACGAATCCCACACCGAATTTG				
*G6PDH*	F: GCAAAGGTGGGCGTATTGGAATC	127	−3.245	0.987	103.314
	R: CAGCACTCACATTGGACGACATG				
*GAPDH*	F: ATAACCTTGCCAACGGCCTTA	93	−3.151	0.998	107.659
	R: TCCCTCCGGCAAACTGT				
*DPH1*	F: CGGCGGTCCAGGAGAACATG	102	−3.494	0.985	93.286
	R: CCAAACGGATCTGACGAACCAAAG				
*Rab1*	F: TGACGATGGCTGCCGAGATAAAG	118	−3.195	0.994	105.581
	R: CAGCAACCTGATTTGGTGTTCTCC				
*RPS13*	F: TTTGAAACCCGACATCCCAGAGG	141	−3.179	0.988	106.331
	R: GCCAAACGGTGGATTCTTGACTC				
*HSP90*	F: TCTTGCGTTACCACACCTCAGC	121	−3.155	0.988	107.471
	R: ACTTGTTCCTTCGACTCACCAGTG				

## Data Availability

The original data are included in this article; further inquiries can be directed to the corresponding author.

## Abbreviations

The following abbreviations are used in this manuscript:

d	Day
*α-tubulin*	Tubulin alpha chain
*G6PDH*	Glucose-6-phosphate dehydrogenase
*Rab1*	Rab1
*RT*	Reverse transcriptase
*RPS13*	40s ribosomal protein s13
*β-tubulin*	Beta-tubulin 1 chain
*DPH1*	DnaJ protein homolog 1
*HSP90*	HSP90A
*GAPDH*	Glyceraldehyde-3-phosphate dehydrogenase 2
*CP*	Cysteine proteinase

## References

[1] Li X., Yang H., Hu K., Wang J. (2020). Temporal dynamics of Bactrocera (Zeugodacus) tau (Diptera: Tephritidae) adults in north Jiangxi, a subtropical area of China revealed by eight years of trapping with cuelure. J. Asia-Pac. Entomol..

[2] Wang Y.T., Cao L.J., Chen J.C., Song W., Ma W.H., Yang J.F., Gao X.Y., Chen H.S., Zhang Y., Tian Z.Y. (2023). Chromosome-level genome assembly of an agricultural pest Zeugodacus tau (Diptera: Tephritidae). Sci. Data.

[3] He Y., Xu Y., Chen X. (2023). Biology, Ecology and Management of Tephritid Fruit Flies in China: A Review. Insects.

[4] Jing L., Chao M.A., Zhe L.I., Bang-Qin Z., Jiang Z., Chao-Liang L., Shuang-Xia J., Hull J.J., Li-Zhen C. (2018). Assessment of suitable reference genes for qRT-PCR analysis in Adelphocoris suturalis. J. Integr. Agric..

[5] Liu X., Zhang Q., Xu W., Yang Y., Fan Q., Ji Q. (2023). The Effect of Cuelure on Attracting and Feeding Behavior in Zeugodacus tau (Walker) (Diptera: Tephritidae). Insects.

[6] Shamshir R.A., Wee S.L. (2024). Comparative Responses of Two Major Cucurbit Pests, Zeugodacus cucurbitae and Zeugodacus tau to Phenylbutanoid Male Lures. J. Chem. Ecol..

[7] Liu P., Li Z., Zhang Q., Qiao J., Zheng C., Zheng W., Zhang H. (2024). Identification of testis development-related genes by combining Iso-Seq and RNA-Seq in Zeugodacus tau. Front. Cell Dev. Biol..

[8] Zhang H.H., Luo M.J., Zhang Q.W., Cai P.M., Idrees A., Ji Q.E., Yang J.Q., Chen J.H. (2019). Molecular characterization of prophenoloxidase-1 (PPO1) and the inhibitory effect of kojic acid on phenoloxidase (PO) activity and on the development of Zeugodacus tau (Walker) (Diptera: Tephritidae). Bull. Entomol. Res..

[9] Bachman J. (2013). Reverse-transcription PCR (RT-PCR). Methods Enzym..

[10] Donovan N.J., Chambers G.A., Cao M. (2022). Detection of Viroids by RT-PCR. Methods Mol. Biol..

[11] Silveira G.O., Amaral M.S., Coelho H.S., Maciel L.F., Pereira A.S.A., Olberg G.G.O., Miyasato P.A., Nakano E., Verjovski-Almeida S. (2021). Assessment of reference genes at six different developmental stages of Schistosoma mansoni for quantitative RT-PCR. Sci. Rep..

[12] Bustin S.A., Benes V., Nolan T., Pfaffl M.W. (2005). Quantitative real-time RT-PCR—A perspective. J. Mol. Endocrinol..

[13] Bustin S.A., Vladimir B., Garson J.A., Jan H., Jim H., Mikael K., Reinhold M., Tania N., Pfaffl M.W., Shipley G.L. (2009). The MIQE Guidelines: Minimum Information for Publication of Quantitative Real-Time PCR Experiments. Clin. Chem..

[14] Vandesompele J., Preter K.D., Pattyn F., Poppe B., Roy N.V., Paepe A.D., Speleman F. (2002). Accurate normalization of real-time quantitative RT-PCR data by geometric averaging of multiple internal control genes. Genome Biol..

[15] Nolan T., Hands R.E., Bustin S.A. (2006). Nature Protocols: Quantification of mRNA using real-time RT-PCR. Nat. Protoc..

[16] Derveaux S., Vandesompele J., Hellemans J. (2010). How to do successful gene expression analysis using real-time PCR—ScienceDirect. Methods.

[17] LourençO A.P., Mackert A., Cristino A.D.S., Sim?es Z.L.P. (2008). Validation of reference genes for gene expression studies in the honey bee, Apis mellifera, by quantitative real-time RT-PCR. Apidologie.

[18] Rumei L., Wen X., Shaoli W., Qingjun W., Nina Y., Xin Y., Huipeng P., Xiaomao Z., Lianyang B., Baoyun X. (2013). Reference Gene Selection for qRT-PCR Analysis in the Sweetpotato Whitefly, Bemisia tabaci (Hemiptera: Aleyrodidae). PLoS ONE.

[19] Shi X.Q., Guo W.C., Wan P.J., Zhou L.T., Ren X.L., Ahmat T., Fu K.Y., Li G.Q. (2013). Validation of reference genes for expression analysis by quantitative real-time PCR in Leptinotarsa decemlineata (Say). BMC Res. Notes.

[20] Miao Y., Yanhui L., Xun Z., Hu W., Muhammad S., Sha Z., Byung-Rae J., Jianhong L., Xiao-Wei W. (2014). Selection and Evaluation of Potential Reference Genes for Gene Expression Analysis in the Brown Planthopper, Nilaparvata lugens (Hemiptera: Delphacidae) Using Reverse-Transcription Quantitative PCR. PLoS ONE.

[21] Volland M., Blasco J., Hampel M. (2017). Validation of reference genes for RT-qPCR in marine bivalve ecotoxicology: Systematic review and case study using copper treated primary Ruditapes philippinarum hemocytes. Aquat. Toxicol..

[22] Shen C.H., Peng L.J., Zhang Y.X., Zeng H.R., Yu H.F., Jin L., Li G.Q. (2022). Reference Genes for Expression Analyses by qRT-PCR in Phthorimaea operculella (Lepidoptera: Gelechiidae). Insects.

[23] Andersen C.L., Jensen J.L., Rntoft T.F. (2004). Normalization of Real-Time Quantitative Reverse Transcription-PCR Data: A Model-Based Variance Estimation Approach to Identify Genes Suited for Normalization, Applied to Bladder and Colon Cancer Data Sets. Am. Assoc. Cancer Res..

[24] Ponton F., Chapuis M.P., Pernice M., Sword G.A., Simpson S.J. (2011). Evaluation of potential reference genes for reverse transcription-qPCR studies of physiological responses in Drosophila melanogaster. J. Insect Physiol..

[25] Shi C.H., Hu J.R., Li C.R., Wang W.K. (2017). Progress in the study of reference genes in insect qRT-PCR. Jiangsu Agric. Sci..

[26] Chen L., Tian Z., Wang X.Y., Chen X.M., Lu W., Wang X.P., Zheng X.L. (2021). Selection of reference genes for real-time fluorescent quantitative PCR in the red-shouldered moth, Euproctis chrysorrhoea. J. Environ. Entomol..

[27] Wolf K.W., Spanel-Borowski K. (1995). Acetylation of α-tubilin in different bovine cell types: Implications for microtubule dynamics in interphase and mitosis. Blackwell Publ. Ltd..

[28] McKenna E.D., Sarbanes S.L., Cummings S.W., Roll-Mecak A. (2023). The Tubulin Code, from Molecules to Health and Disease. Annu. Rev. Cell Dev. Biol..

[29] Moraes B., Martins R., Lopes C., Martins R., Arcanjo A., Nascimento J., Konnai S., Itabajara D.S.V.J., Logullo C. (2023). G6PDH as a key immunometabolic and redox trigger in arthropods. Front. Physiol..

[30] Liu T. (2021). Evaluation of Reference Genes for Quantitative Reverse Transcription Polymerase Chain Reaction in Bactrocera dorsalis (Diptera: Tephritidae) Subjected to Various Phytosanitary Treatments. Insects.

[31] Zhou X., Liao W.J., Liao J.M., Liao P., Lu H. (2015). Ribosomal proteins: Functions beyond the ribosome. J. Mol. Cell Biol..

[32] Lü J., Yang C., Zhang Y., Pan H. (2018). Selection of Reference Genes for the Normalization of RT-qPCR Data in Gene Expression Studies in Insects: A Systematic Review. Front. Physiol..

[33] Shen X.X., Zhang G.Q., Zhao Y.X., Zhu X.X., Yu X.F., Yang M.F., Zhang F. (2023). Selection and validation of optimal reference genes for RT-qPCR analyses in Aphidoletes aphidimyza Rondani (Diptera: Cecidomyiidae). Front. Physiol..

[34] Neuman S.D., Lee A.R., Selegue J.E., Cavanagh A.T., Bashirullah A. (2021). A novel function for Rab1 and Rab11 during secretory granule maturation. J. Cell Sci..

[35] Zhang J., Jiang Z., Shi A. (2022). Rab GTPases: The principal players in crafting the regulatory landscape of endosomal trafficking. Comput. Struct. Biotechnol. J..

[36] Sechi S., Frappaolo A., Fraschini R., Capalbo L., Giansanti M.G. (2017). Rab1 interacts with GOLPH3 and controls Golgi structure and contractile ring constriction during cytokinesis in Drosophila melanogaster. Open Biol..

[37] Lee J., Kim Y.H., Kim K., Kim D., Lee S.H., Kim S. (2022). Selection of stable reference genes for quantitative real-time PCR in the Varroa mite, Varroa destructor. Arch. Insect Biochem. Physiol..

[38] He Q.Y., Yan J.Y., Zhou Y.T. (2024). Selection and Verification of the Referwnce Genes in lliberis pruni (Lepidoptera: Zygaenidae). J. Gansu Agric. Univ..

[39] Lü Z.C., Wang L.H., Dai R.L., Wan F.H. (2014). Evaluation of Endogenous Reference Genes of Bactrocera (Tetradacus) minax by Gene Expression Profiling under Various Experimental Conditions. Fla. Entomol. Soc..

